# Tumor infiltrating lymphocytes (TILs) as a predictive marker of pathological complete response (pCR) in a diverse patient population with early triple negative breast cancer (TNBC) treated with neoadjuvant real-world KEYNOTE-522 regimen

**DOI:** 10.1007/s10549-026-08002-7

**Published:** 2026-06-26

**Authors:** Riya Albert, Joshua Thomas, Navid Sadeghi, Sangeetha M. Reddy, Glenda Delgado Ramos, Heather McArthur, Samira Syed, Deborah Farr, Nisha Unni

**Affiliations:** 1 UT Southwestern Medical Center, Dallas, USA; 2 University of Texas Austin, Austin, USA; 3 UT Southwestern Simmons Comprehensive Cancer Center, Dallas, USA

**Keywords:** Tumor-infiltrating lymphocytes, Triple-negative breast cancer, Neoadjuvant therapy, Immunotherapy, Pathological complete response

## Abstract

**Introduction:**

Triple-negative breast cancer (TNBC) is an aggressive subtype characterized by poor prognosis. Based on the KEYNOTE-522 trial, neoadjuvant pembrolizumab plus chemotherapy has become the standard of care due to significantly improved pathological complete response (pCR) rates. The presence of tumor-infiltrating lymphocytes (TILs) is a predictive biomarker of pCR. This retrospective cohort study examines a diverse patient population treated with the K522 regimen to determine if TILs predict pCR relative to other clinical and tumor-specific factors.

**Methods:**

We retrospectively reviewed 187 patients with early-stage TNBC at two institutions (one tertiary care, one safety-net) who completed neoadjuvant K522 treatment between 2021 and 2024. Statistical analyses included Chi-squared tests, Z-tests, and univariate logistic regression to evaluate associations between TILs, ethnicity, tumor grade, and pCR, and multivariate logistic regression to assess whether the association between dose intensity and pCR differed by the presence of TILs at baseline.

**Results:**

The overall pCR rate was 57%; TILs were present in 52.8% of cases. TILs were associated with a significantly higher pCR rate (70% vs. 48% without TILs; *p* = 0.0027). While pCR rates were similar across ethnicities, Hispanic patients with TILs had significantly higher pCR than those without (80.0% vs. 51.5%; *p* = 0.0254). Controlling for grade, patients with TILs were 2.442 times more likely to achieve pCR (CI: 1.310–4.553; *p* = 0.0050). Grade 3 tumors and node-positivity with TILs also showed statistically significant rates of pCR. Logistic regression analysis revealed a significant interaction between TIL status and immunotherapy dose intensity on pCR. While dose intensity did not influence pCR in TIL-negative patients, TIL-positive patients achieved higher pCR rates with lower dose intensity (OR = 1.54, *p* = 0.036).

**Conclusion:**

TILs serve as a strong predictive biomarker for immunotherapy response in a real-world TNBC population. Our findings regarding Hispanic, node-positive patients, and the interaction between TIL status and dose intensity suggest that TILs could guide treatment de-escalation to reduce K522-related toxicity. Standardizing TILs reporting is critical to optimizing treatment strategies and improving outcomes in underrepresented populations.

## Introduction

Triple-negative breast cancer (TNBC) is an aggressive subtype of breast cancer defined by its lack of estrogen receptors (ER), progesterone receptors (PR), and HER2/neu. It accounts for 15% of all breast cancer diagnoses, yet due to the lack of typical receptor biomarkers, there are limited effective targeted therapies in early-stage disease. Patients with TNBC tend to present with higher-stage disease and generally have worse prognoses than other breast cancer subtypes. Previously, the standard practice for treatment was neoadjuvant chemotherapy (NAC), using anthracycline- and taxane-based regimens.

In July 2021, based on results from the KEYNOTE-522 (K522) trial, the FDA approved pembrolizumab (Keytruda) with chemotherapy as neoadjuvant therapy for high-risk early-stage TNBC, followed by adjuvant monotherapy after surgery, which significantly improved prognosis [1, 2]. The K522 trial examined rates of pathologic complete response (pCR) as a preliminary efficacy outcome measure due to its association with good prognosis and long-term disease-free survival [3]. Compared with neoadjuvant chemotherapy alone, the K522 regimen was associated with a higher pCR rate (64.8% vs. 51.2%) and a clinically significant improvement in event-free survival at 60 months (86.6% vs. 81.7%).

Despite these promising improvements in outcomes, response to neoadjuvant therapy with the K522 regimen in early-stage TNBC is still variable, and our understanding of what underlying characteristics may be associated with successful treatment response is limited. Investigating the clinical and pathology-based variables linked to pCR following the K522 regimen may help identify predictive factors for anticipating treatment response.

The presence of tumor-infiltrating lymphocytes (TILs) has been identified as a predictive biomarker for TNBC, as high levels of TILs are associated with better overall survival and disease-free survival [4]. Previous studies correlate high TIL levels with better response to NAC, especially in aggressive subtypes, such as TNBC [5, 6]. However, the extent to which TILs predict response to neoadjuvant immunotherapy in early-stage TNBC, particularly in comparison to other tumor and clinical factors, remains unclear. To investigate the predictive value of TILs in early-stage TNBC, we conducted a retrospective cohort study in a clinically diverse patient population who received the K522 regimen to determine whether the presence of TILs confers a predictive value in response to neoadjuvant pembrolizumab treatment compared to various other tumor and clinical factors.

## Methods

### Patient selection

We reviewed electronic medical records of 271 early-stage TNBC patients who received neoadjuvant treatment in accordance with the KEYNOTE-522 regimen at an NCI-designated comprehensive cancer center, UT Southwestern Harold C. Simmons Comprehensive Cancer Center (UTSW-SCCC), and its affiliated safety-net hospital, Parkland Health (PH), between August 2021 to December 2024. Using retrospective chart review from both institutions, we identified 184 patients who completed the neoadjuvant portion of the study and underwent surgery at the time of chart review. Patients were excluded if they had metastatic disease at the time of diagnosis, did not proceed with surgery due to disease progression or adverse events, or had not undergone surgery at the time of data collection.

This study was approved by the Institutional Review Board (IRB) of UT Southwestern Medical Center and Parkland Health.

Data was collected from each institution through medical record review and included the following details: patient ID, site, age at diagnosis, date of birth (DOB), race, ethnicity, tumor type, tumor stage (T and N), neoadjuvant chemotherapy regimen, number of pembrolizumab cycles, presence of TILs indicated in the baseline biopsy report, post-surgery tumor characteristics (ypT, ypN, residual cancer burden [RCB]), hormone receptor status (estrogen receptor [ER] and progesterone receptor [PR] expression at diagnosis and on surgical samples, reported as percentage of tumor cell nuclei staining positive via immunohistochemistry), HER2 status (by IHC and FISH at diagnosis and on surgical samples), tumor grade, Ki-67 proliferation index at diagnosis and on surgical samples, history of autoimmune disease, and additional notes.

TNBC was defined as the absence of estrogen receptor (ER) and progesterone receptor (PR) expression (< 10%) and a HER2-negative status (score of 0+, 1+, or 2 + with a confirmatory non-amplified result from fluorescent in situ hybridization), in accordance with the most recent American Society of Clinical Oncology/College of American Pathologists (ASCO/CAP) guidelines [7].

RCB ranged from RCB 0, indicating pCR, to RCB III, indicating extensive residual disease. The classifications were determined using MD Anderson’s web calculator for residual cancer burden. pCR was defined as the lack of residual invasive carcinoma in both breast tumor bed tissue and axillary lymph nodes found at the time of surgical resection following neoadjuvant therapy, with final pathologic stages as ypT0/Tis and ypN0.

### Immunohistochemistry and TILs assessment

**Fig. 1:**
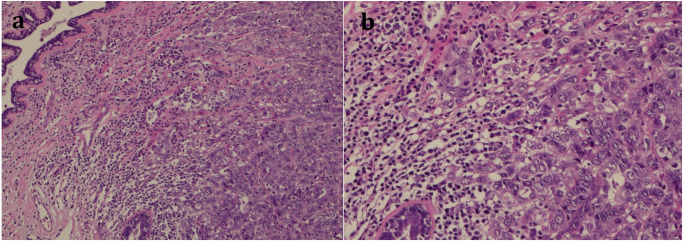
Example pathology slide of TILs in TNBC patient low (**a**) and high power (**b**)

Tumor-infiltrating lymphocytes (TILs) were evaluated in formalin-fixed, paraffin-embedded (FFPE) tumor tissue samples stained with hematoxylin and eosin (H&E) from baseline biopsies, in accordance with the guidelines established by the International TILs Working Group [8] (Fig. 1). TILs were assessed in both intratumoral regions (invasive tumor nests, including intraepithelial areas) and stromal regions (intratumoral stroma. The evaluation prioritized intratumoral stromal TILs, followed by peripheral stromal TILs, particularly within 1–2 mm of the tumor-stroma interface. Both regions were included in the final TILs assessment, with greater emphasis placed on intratumoral stromal TILs.

Based on the guidelines of the International TILs Working Group, TILs were quantified as a percentage of the total stromal or intratumoral area occupied by mononuclear cells (including lymphocytes and plasma cells) [8]. While TILs are typically reported in 10% increments, pathologists also estimated percentages in smaller increments when necessary. TILs were considered significant when they constituted approximately 25–30% or more of the assessed area.

### Statistical analysis

Statistical analysis was conducted to evaluate the association between pathological complete response (pCR) rates and various demographic and clinical factors, including tumor-infiltrating lymphocytes (TILs), tumor grade, and residual cancer burden (RCB). Two-proportion Z-tests and Chi-squared tests for independence were employed to compare pCR rates across different demographic groups, including ethnicity and the presence or absence of TILs. Logistic regression models were used to assess the relationship between the presence of TILs, tumor grade, and RCB with pCR outcomes. Additionally, multivariable logistic regression models were used to evaluate the association between dose intensity of neoadjuvant therapy and odds of achieving pCR. An interaction term between dose intensity and TIL status was included to assess whether the association between dose intensity and pCR differed according to the baseline presence of TILs.

## Results

The median age of our patient population was 51 years, with an age range of 24 to 83 years. Of which, 34.8% self-identified as Caucasian (C), 31.0% Hispanic (H), 25.5% Black (B), and 8.7% other. All patients were female. Patient demographics, tumor type, size (T), and nodal status (N) are shown in Table 1. Along with immunotherapy, most patients received an anthracycline/taxane-based chemotherapy regimen, starting with four cycles of paclitaxel and carboplatin followed by another four cycles of doxorubicin and cyclophosphamide. 68.6% of patients completed 8 or more cycles of neoadjuvant therapy, and 81.5% of patients completed at least 75% of treatment. Among patients who completed less than 8 cycles, 81.1% discontinued due to adverse reactions.

**Table 1:** Patient demographics and clinical characteristics

Patient Demographics		
*n* = 184		
***Parameters***	***Median***	***SD***
Age (Years)	*51*	*13.6*
BMI (kg/m^2^)	*30.1*	*6.8*
	***n (%)***	
Ethnicity		
White	*64 (34.8)*	
Black	*46 (25.0)*	
Hispanic	*57 (30.1)*	
Asian/Other	*16 (8.7)*	
***Tumor Characteristics***		
Type		
Ductal	*171 (92.9)*	
Lobular	*5 (2.7)*	
Mixed	*8 (4.4)*	
Grade		
1	*0 (0.0)*	
2	*28 (15.2)*	
3	*156 (84.8)*	
Tumor Classification (T)		
T1,2	*139 (75.5)*	
T3,4	*45 (24.5)*	
Nodal Status (N)		
Negative	*83 (45.1)*	
Positive	*101 (54.9)*	
**Response to Treatment**		
Pathological Response		
RCB 0 (pCR)	*105 (57.1)*	
RCB 1	*12 (6.5)*	
RCB 2	*42 (22.8)*	
RCB 3	*25 (13.6)*	

### Pathological complete response rate in subpopulations

Overall, 57% of patients achieved a pathological complete response (pCR). TILs were present and reported in 52.8% of pathology reports. We did not see an increased presence of TILs in any ethnic population (χ² = 2.5806, *p* = 0.4609).

Univariate logistic regression models were used to evaluate the association between age, ethnicity, tumor stage, tumor grade, and presence of TILs, with pCR (RCB 0) versus residual disease (RCB 1, 2, 3). For a secondary analysis, responses were grouped as RCB 0, 1 versus RCB 2, 3 to further assess potential associations, as shown in Table 2. Tumor grade and the presence of TILs were the only variables significantly associated with pCR in the first grouping; however, only TILs remained significant in the second grouping. Specifically, the presence of TILs was associated with increased pCR rates compared to those without TILs (*69.6*% vs. *49.1%)*, which was statistically significant by univariate analysis *(p* = 0.0027) and Chi-squared test (χ² = 10.75, *p* = 0.013).

Within ethnic subpopulations, Hispanic patients with TILs demonstrated a higher pCR rate (80.0%) compared to those without TILs (51.5%) (*p* = 0.0254). Although a trend toward significance was observed, the sample was underpowered to detect a definitive association. No other ethnic group demonstrated an association between the presence of TILs and pCR.

Univariate logistic regression models were used to evaluate associations between ethnicity, tumor stage, nodal status, tumor grade, presence of TILs, RCB, and pCR. Only tumor grade and the presence of TILs were significantly associated with pCR. Patients with grade 3 tumors were 2.89 times more likely to achieve pCR than those with lower-grade disease (95% CI: 1.28–11.82; *p* = 0.017), and higher tumor grade remained associated with increased odds of pCR in a separate model comparing grade 3 to grade 2 (OR = 2.76; 95% CI: 0.998–7.62; *p* = 0.0505). After adjusting for tumor grade, patients with TILs were 2.44 times more likely to achieve pCR (95% CI: 1.31–4.55; *p* = 0.005). A model incorporating both TILs and tumor grade demonstrated moderate predictive performance, with an AUC of 0.64 (95% CI: 0.562–0.708).

The ROC curve for the stepwise logistic regression model, which included TILs and tumor grade, demonstrated moderate predictive performance, with an AUC of 0.64 (95% CI: 0.562–0.708).

**Table 2:** pCR (RCB = 0) rates in subpopulations

	pCR (RCB = 0)	RD (RCB ≥ 1)	All	*p* Value
	*n* = 108	*n* = 79	*n* = 184	
**Patient Demographics**				
Age at diagnosis—x̄±s	*49.6 ± 11.2*	*53.3 ± 12.6*	*51 ± 11.9*	***0.035***
Ethnicity—No. (%)				*0.465*
Asian	*7 (43.8)*	*9 (56.3)*	*16 (8.6)*	
Black	*25 (53.2)*	*22 (46.8)*	*47 (25.3)*	
Hispanic	*37 (63.8)*	*21 (36.2)*	*58 (31.8)*	
White	*38 (58.5)*	*27 (41.5)*	*65 (34.9)*	
Hispanic—No. (%)				*0.245*
Hispanic	*37 (63.8)*	*21(36.2)*	*58 (31.2)*	
Non-Hispanic	*70 (54.7)*	*58 (45.3)*	*128 (68.8)*	
White—No. (%)				*0.850*
Non-White	*69 (57.0)*	*52 (43.0)*	*121 (65.1)*	
White	*38 (58.5)*	*27 (41.5)*	*65 (34.9)*	
**Tumor Characteristics**				
Tumor Classification (T)—No. (%)				*0.565*
T 1,2	*76 (58.9)*	*53 (41.1)*	*129 (69.4)*	
T 3,4	*31 (54.4)*	*26 (45.6)*	*57 (30.7)*	
Nodal Status (N)—No. (%)				*0.703*
Negative	*53 (56.4)*	*41 (43.6)*	*94 (50.3)*	
Positive	*55 (59.1)*	*38 (40.9)*	*93 (49.7)*	
TILs present—No. (%)				***0.005***
Absent	*53 (49.1)*	*55 (50.9)*	*108 (57.8)*	
Present	*55 (69.6)*	*24 (30.4)*	*79 (42.2)*	
Grade—No. (%)				***0.013***
2	*5 (29.4)*	*12 (70.6)*	*17 (9.1)*	
3	*103 (60.1)*	*67 (39.4)*	*170 (90.9)*	
**TILs by Ethnicity**				
Hispanic)—No. (%) *n* = 57				***0.025***
TILs present	*20 (80.0)*	*5 (20.0)*	*25 (39.7)*	
TILs absent	*17 (51.5)*	*16 (48.5)*	*33 (60.3)*	
Black)—No. (%) *n* = 46				*0.096*
TILs present	*14 (66.7)*	*7 (33.3)*	*21 (45.7)*	
TILs absent	*11 (16.9)*	*15 (83.1)*	*65 (54.3)*	
White)—No. (%) *n* = 64				*0.214*
TILs present	*20 (66.7)*	*10 (33.3)*	*30 (46.9)*	
TILs absent	*18 (51.4)*	*17 (48.6)*	*35 (53.1)*	
Asian—No. (%) *n* = 17				*0.671*
TILs present	*2 (50)*	*2 (50)*	*4 (23.5)*	
TILs absent	*5 (41.7)*	*7 (58.3)*	*12 (76.5)*	

### Node positivity and association with pCR in patients with TILs

Within the TIL population, node positivity (N) was associated with statistically significant improvement in pCR rates (*p* = 0.0112). This association was further pronounced when these patients were regrouped into RCB 0, 1 and RCB 2, 3 (*p* = 0.0051). However, the presence of TILs was not associated with improvement in pCR in node-negative patients regardless of grouping (RCB 0 vs. 1, 2, 3: *p* = 0.1471 and RCB 0,1 vs. 2, 3: *p* = 0.1567).

### Association between dose intensity and pCR in patients based on TILs status

On logistic regression analysis, we found a correlation based on dose intensity and pCR in the presence and absence of TILs. In the absence of TILs, additional doses of neoadjuvant chemoimmunotherapy did not alter the odds of achieving pCR (OR = 1.07, *p* = 0.57). However, when TILs were present, additional doses of chemoimmunotherapy were associated with reduced odds of achieving pCR (OR = 0.7, *p* = 0.026). Those who received fewer doses (< 5) had higher pCR rates (75%) whereas those who received more doses had lower pCR rates (12.5–22.2%). The interaction term between the two logistic regressions was also significant, confirming that the effect of dose intensity on the odds of achieving pCR significantly differs depending on whether TILs are present or absent (OR = 1.54, *p* = 0.036).

## Discussion

In this real-world retrospective analysis, the presence of TILs in early-stage TNBC patients demonstrated a strong predictive value for achieving pCR to the K522 regimen. This association was particularly pronounced in specific subpopulations, with TILs more strongly correlated with pCR in Hispanic patients and patients with positive nodal involvement. Our findings are consistent with the results of the K522 trial and suggest potential subgroups in which the role of TILs may warrant further investigation as predictors of treatment response.

Previous studies have demonstrated that pCR rates to neoadjuvant chemo-immunotherapy in TNBC are overall higher than with chemotherapy alone, yet response varies by patient demographics. In the original K522 trial, addition of pembrolizumab significantly improved pCR rates (64.8%) compared to chemotherapy alone (51.2%).^1^ However, secondary subgroup analyses demonstrated lower efficacy in different study populations, specifically patients enrolled in Asian countries [9]. Likewise, analyses of real-world applications of the K522 regimen have also reported lower pCR rates than those seen in the controlled trial setting [9–12]. Prior research on neoadjuvant chemotherapy response has shown that Black patients with TNBC have lower pCR rates [13], and that older age is associated with reduced treatment response [12, 14]. Our study mirrors these real-world findings, reporting a higher overall pCR rate with pembrolizumab, though slightly lower (57%) than that observed in the original trial. This difference may be attributable to the demographic composition of our cohort, which included a higher proportion of Hispanic and Black patients and an older median age. While our sample size was not sufficient to detect statistically significant differences across subgroups, these results reinforce the need to consider demographic variability when evaluating immunotherapy responses in real-world settings. Nevertheless, the relative benefit of the K522 regimen was seen across all ethnicities.

While the K522 trial did not evaluate TILs in relation to immunotherapy response, growing evidence highlights their role as a surrogate of the host anti-tumor immune response. Their presence has been associated with favorable outcomes in both treated and untreated TNBC [15]. The International TILs Working Group established a standardized scoring system with high interobserver reproducibility, supporting TILs as a viable and standardizable biomarker [8]. Several studies evaluated the predictive value of TILs in the context of chemotherapy. For example, a pooled analysis showed that patients with primary TNBC treated with NAC had a stepwise increase in pCR with increasing TILs, as well as improved disease-free and overall survival with incremental increase in TIL density [15]. Although the predictive role of TILs in response to immunotherapy is less defined, emerging data are encouraging. In metastatic TNBC, biomarker analyses from the KEYNOTE-086 and KEYNOTE-119 trials showed associations between TIL levels and both treatment response and overall outcomes with single-agent pembrolizumab [16, 17]. In early-stage disease, although the GeparNuevo trial found that TILs predicted higher pCR rates in both neoadjuvant immunotherapy and placebo arms, a dynamic increase in TIL density was independently associated with increased pCR in the immunotherapy group alone [18]. Furthermore, the KEYNOTE-173 and I-SPY2 trials demonstrated correlations between higher baseline TILs and pCR after treatment with pembrolizumab and NAC [19, 20]. Similar to these prior studies, our findings also demonstrated a significant association between TILs and increased pCR. Collectively, these data support the potential use of TILs as a predictive marker of response in early TNBC.

The predictive significance of TILs was particularly pronounced in Hispanic patients within our cohort. We speculate that this enhanced predictive value of TILs may be related to underlying diversity in genetic ancestry and allelic predisposition. One well-characterized example of a population-based polymorphism is the human leukocyte antigen (HLA) gene. Greater heterozygosity of HLA alleles has been associated with more effective T-cell response to malignancies, higher density of TILs, and better outcomes with immunotherapy, likely due to the presentation of a broader range of tumor antigens. Notably, Hispanics in the United States. have been found to have increased allelic heterozygosity at the HLA locus, reflecting a complex genetic ancestry [21, 22]. This pronounced genetic diversity, particularly regarding HLA polymorphism, may contribute directly to the strong association observed between baseline TILs and treatment response in our Hispanic cohort. These findings highlight the critical importance of integrating genetic ancestry into biomarker development and immunotherapy research.

In the original K522 trial, patients with node-positive disease or with high-grade (grade 3) tumors had higher pCR rates with the addition of pembrolizumab compared to those with low-stage or grade disease, suggesting that tumor burden may enhance immunotherapy responsiveness.^1^ Our findings also demonstrated that high-grade tumors had increased pCR rates with the K522 regimen. In addition, for patients with node-positive disease, the presence of TILs was predictive of pCR. Lymph node involvement reflects more aggressive tumor biology, characterized by higher mutational burden, and promotes TIL recruitment and immune activation. Our study confirms and extends these findings, as the predictive value of TILs was more pronounced in patients with node-positive disease. Patients with high-risk features such as high tumor stage or grade have a higher baseline risk of recurrence, therefore the relationship between TILs and pCR may have prognostic value for long-term outcomes in these patients.

Our analysis also reveals a statistically significant interaction between baseline TIL status and neoadjuvant chemoimmunotherapy dose intensity on pCR rates (*p* = 0.036), demonstrating that TILs could function as a predictive biomarker for optimizing the duration and intensity of neoadjuvant therapy. Increase in cumulative exposure to chemoimmunotherapy may impair the tumor microenvironment, decrease immune responsiveness and reduce TIL activity, therefore, may result in decreased pCR rates in these patients. Prior studies have demonstrated a significant reduction in TIL density and FoxP3 expression within the tumor microenvironment following neoadjuvant chemotherapy [23, 24]. Chemotherapy has been shown to exert a biphasic immunomodulatory effect, characterized by an initial phase of immune activation succeeded by progressive immunosuppression, ultimately diminishing overall immune responsiveness. Consequently, the observed inverse relationship between TIL presence and both dose intensity and pathological complete response likely reflects chemotherapy-mediated immune modulation rather than an effect attributable to immunotherapy. Given the well-documented toxicities of aggressive neoadjuvant chemoimmunotherapy, using TILs as biomarkers to tailor treatment duration could be a crucial step toward avoiding overtreatment, reducing adverse events, and improving outcomes across patient populations.

## Conclusion

In conclusion, the findings in this study demonstrate a promising predictive value of TILs in a diverse, real-world patient population receiving the K522 regimen. The ability of TILs to predict response to immunotherapy, especially within specific sub-populations, may provide guidance to de-escalate neoadjuvant therapy for these patients.

Given the high potential for toxicity with the K522 regimen, identifying patients likely to respond to de-escalated treatment based on immune biomarkers could help avoid overtreatment and reduce unnecessary adverse events. Establishing TILs as a predictive marker could not only enhance patient stratification for tailored therapies but also lead to improved outcomes in diverse populations affected by early-stage TNBC.

While these findings are promising, they should be interpreted within the context of our study’s limitation. Our study was underpowered to detect definitive associations within subpopulations, although certain groups demonstrated trends toward significance. Larger, targeted studies focusing on Hispanic patients and those with node-positive TNBC are warranted to better understand the predictive value of TIL in this population.

Our sample size was also not powered to detect interaction effects, and the observed estimates about the relationship between dose intensity and pCR in TIL positive tumors should be interpreted cautiously. However, the demonstration of a statistically significant interaction despite limited power is promising and suggests an underlying biologically relevant association. These findings should be considered hypothesis-generating and require validation in larger, independently powered cohorts. Furthermore, our statistical analysis excluded patients who experienced disease progression during neoadjuvant therapy. Incorporating this cohort in future analyses could reveal an even greater difference in treatment efficacy, potentially further favoring patients with robust baseline TIL expression.

Finally, TILs data were extracted from pathology reports, and biopsy slides were not independently reviewed for purposes of TILs identification or quantification. The presence of TILS is routinely documented in pathology reports at our institution. More nuanced information on the quantity of TILs would be more informative; however, quantitative assessment based on the standardized criteria established by the International TILs Working Group is not part of routine practice. Furthermore, our analyses did not account for specific characteristics of TILs such as variations in location and functional activity, which may also influence treatment response. We recommend further study of the predictive value of TILs based on density and specific histopathologic features. This information may highlight clinically meaningful thresholds that more specifically correlate with treatment response. Current College of American Pathologists guidelines do not mandate standardized reporting of TILs in pathology reports. However, to fully assess the potential of TILs as a predictive marker in early-stage TNBC, future prospective studies that incorporate standardized reporting are critical, especially to explore the role of TILs in guiding de-escalation strategies for chemotherapy.

## Data Availability

No datasets were generated or analysed during the current study.

## References

[1] Schmid P, Cortes J, Pusztai L et al (2020) Pembrolizumab for Early Triple-Negative Breast Cancer. N Engl J Med 382:810–821. 10.1056/NEJMoa1910549 32101663 10.1056/NEJMoa1910549

[2] Schmid P, Cortes J, Dent R et al (2024) Overall Survival with Pembrolizumab in Early-Stage Triple-Negative Breast Cancer. N Engl J Med 391:1981–1991. 10.1056/NEJMoa2409932 39282906 10.1056/NEJMoa2409932

[3] Cortazar P, Zhang L, Untch M et al (2014) Pathological complete response and long-term clinical benefit in breast cancer: the CTNeoBC pooled analysis. Lancet 384:164–172. 10.1016/S0140-6736(13)62422-8 24529560 10.1016/S0140-6736(13)62422-8

[4] Huertas-Caro CA, Ramírez MA, Rey-Vargas L et al (2023) Tumor infiltrating lymphocytes (TILs) are a prognosis biomarker in Colombian patients with triple negative breast cancer. Sci Rep 13:21324. 10.1038/s41598-023-48300-4 38044375 10.1038/s41598-023-48300-4 PMC10694133

[5] Ruan M, Tian T, Rao J, Xu X, Yu B, Yang W, Shui R (2018) Predictive value of tumor-infiltrating lymphocytes to pathological complete response in neoadjuvant-treated triple-negative breast cancers. Diagn Pathol 13(1):66. 10.1186/s13000-018-0743-7 10.1186/s13000-018-0743-7 PMC6119339 30170605

[6] Eryilmaz MK, Mutlu H, Ünal B et al (2018) The importance of stromal and intratumoral tumor lymphocyte infiltration for pathologic complete response in patients with locally advanced breast cancer. J Cancer Res Ther 14:619–624. 10.4103/0973-1482.174550 29893329 10.4103/0973-1482.174550

[7] Wolff AC, Somerfield MR, Dowsett M et al (2023) Human Epidermal Growth Factor Receptor 2 Testing in Breast Cancer: ASCO-College of American Pathologists Guideline Update. J Clin Oncol 41:3867–3872. 10.1200/JCO.22.02864 37284804 10.1200/JCO.22.02864

[8] Salgado R, Denkert C, Demaria S et al (2015) The evaluation of tumor-infiltrating lymphocytes (TILs) in breast cancer: recommendations by an International TILs Working Group 2014. Ann Oncol 26:259–271. 10.1093/annonc/mdu450 25214542 10.1093/annonc/mdu450 PMC6267863

[9] Takahashi M, Cortés J, Dent R et al (2023) Pembrolizumab Plus Chemotherapy Followed by Pembrolizumab in Patients With Early Triple-Negative Breast Cancer: A Secondary Analysis of a Randomized Clinical Trial. JAMA Netw Open 6:e2342107. 10.1001/jamanetworkopen.2023.42107 37966841 10.1001/jamanetworkopen.2023.42107 PMC10652156

[10] Wood SJ, Gao Y, Lee J-H et al (2024) High tumor infiltrating lymphocytes are significantly associated with pathological complete response in triple negative breast cancer treated with neoadjuvant KEYNOTE-522 chemoimmunotherapy. Breast Cancer Res Treat 205:193–199. 10.1007/s10549-023-07233-2 38286889 10.1007/s10549-023-07233-2

[11] Connors C, Valente SA, ElSherif A et al (2025) Real-World Outcomes with the KEYNOTE-522 Regimen in Early-Stage Triple-Negative Breast Cancer. Ann Surg Oncol 32:912–921. 10.1245/s10434-024-16390-7 39436619 10.1245/s10434-024-16390-7 PMC11843215

[12] LeVee A, Wong M, Flores S et al (2024) Impact of neoadjuvant pembrolizumab adherence on pathologic complete response in triple-negative breast cancer: a real-world analysis. Oncologist 29:566–574. 10.1093/oncolo/oyae064 38656345 10.1093/oncolo/oyae064 PMC11224989

[13] Woriax HE, Thomas SM, Plichta JK et al (2024) Racial/Ethnic Disparities in Pathologic Complete Response and Overall Survival in Patients With Triple-Negative Breast Cancer Treated With Neoadjuvant Chemotherapy. J Clin Oncol 42:1635–1645. 10.1200/JCO.23.01199 38394476 10.1200/JCO.23.01199 PMC11095870

[14] von Waldenfels G, Loibl S, Furlanetto J et al (2018) Outcome after neoadjuvant chemotherapy in elderly breast cancer patients - a pooled analysis of individual patient data from eight prospectively randomized controlled trials. Oncotarget 9:15168–15179. 10.18632/oncotarget.24586 29632634 10.18632/oncotarget.24586 PMC5880594

[15] El Bairi K, Haynes HR, Blackley E et al (2021) The tale of TILs in breast cancer: A report from The International Immuno-Oncology Biomarker Working Group. npj Breast Cancer 7:1–17. 10.1038/s41523-021-00346-1 34853355 10.1038/s41523-021-00346-1 PMC8636568

[16] Loi S, Adams S, Schmid P et al (2017) Relationship between tumor infiltrating lymphocyte (TIL) levels and response to pembrolizumab (pembro) in metastatic triple-negative breast cancer (mTNBC): Results from KEYNOTE-086. Ann Oncol 28:v608. 10.1093/annonc/mdx440.005

[17] Loi S, Winer E, Lipatov O et al (2020) Relationship between tumor-infiltrating lymphocytes (TILs) and outcomes in the KEYNOTE-119 study of pembrolizumab vs chemotherapy for previously treated metastatic triple-negative breast cancer (mTNBC). Cancer Res 80

[18] Loibl S, Untch M, Burchardi N et al (2019) A randomised phase II study investigating durvalumab in addition to an anthracycline taxane-based neoadjuvant therapy in early triple-negative breast cancer: clinical results and biomarker analysis of GeparNuevo study. Ann Oncol 30:1279–1288. 10.1093/annonc/mdz158 31095287 10.1093/annonc/mdz158

[19] Schmid P, Salgado R, Park YH et al (2020) Pembrolizumab plus chemotherapy as neoadjuvant treatment of high-risk, early-stage triple-negative breast cancer: results from the phase 1b open-label, multicohort KEYNOTE-173 study. Ann Oncol 31:569–581. 10.1016/j.annonc.2020.01.072 32278621 10.1016/j.annonc.2020.01.072

[20] Leon-Ferre RA, Jonas SF, Salgado R et al (2024) Tumor-Infiltrating Lymphocytes in Triple-Negative Breast Cancer. JAMA 331:1135–1144. 10.1001/jama.2024.3056 38563834 10.1001/jama.2024.3056 PMC10988354

[21] Ellis JM, Henson V, Slack R et al (2000) Frequencies of HLA-A2 alleles in five U.S. population groups: Predominance of A∗02011 and identification of HLA-A∗0231. Hum Immunol 61:334–340. 10.1016/S0198-8859(99)00155-X 10689125 10.1016/s0198-8859(99)00155-x

[22] Arrieta-Bolaños E, Hernández-Zaragoza DI, Barquera R (2023) An HLA map of the world: A comparison of HLA frequencies in 200 worldwide populations reveals diverse patterns for class I and class II. Front Genet 14:866407. 10.3389/fgene.2023.866407 37035735 10.3389/fgene.2023.866407 PMC10076764

[23] Llano-León M, Martínez-Enriquez LC, Rodríguez-Bohórquez OM et al (2023) Effect of neoadjuvant chemotherapy on tumor immune infiltration in breast cancer patients: Systematic review and meta-analysis. PLoS ONE 18:e0277714. 10.1371/journal.pone.0277714 37104271 10.1371/journal.pone.0277714 PMC10138237

[24] Mittendorf EA, Zhang H, Barrios CH et al (2020) Neoadjuvant atezolizumab in combination with sequential nab-paclitaxel and anthracycline-based chemotherapy versus placebo and chemotherapy in patients with early-stage triple-negative breast cancer (IMpassion031): a randomised, double-blind, phase 3 trial. Lancet 396:1090–1100. 10.1016/S0140-6736(20)31953-X 32966830 10.1016/S0140-6736(20)31953-X

